# An Overview of Electron Acceptors in Microbial Fuel Cells

**DOI:** 10.3389/fmicb.2017.00643

**Published:** 2017-04-19

**Authors:** Deniz Ucar, Yifeng Zhang, Irini Angelidaki

**Affiliations:** ^1^Department of Environmental Engineering, Harran UniversitySanliurfa, Turkey; ^2^GAP Renewable Energy and Energy Efficiency Center, Harran UniversitySanliurfa, Turkey; ^3^Department of Environmental Engineering, Technical University of DenmarkLyngby, Denmark

**Keywords:** microbial fuel cell, cathodic electron acceptors, cathodic reaction, electricity production, renewable energy, wastewater treatment

## Abstract

Microbial fuel cells (MFC) have recently received increasing attention due to their promising potential in sustainable wastewater treatment and contaminant removal. In general, contaminants can be removed either as an electron donor via microbial catalyzed oxidization at the anode or removed at the cathode as electron acceptors through reduction. Some contaminants can also function as electron mediators at the anode or cathode. While previous studies have done a thorough assessment of electron donors, cathodic electron acceptors and mediators have not been as well described. Oxygen is widely used as an electron acceptor due to its high oxidation potential and ready availability. Recent studies, however, have begun to assess the use of different electron acceptors because of the (1) diversity of redox potential, (2) needs of alternative and more efficient cathode reaction, and (3) expanding of MFC based technologies in different areas. The aim of this review was to evaluate the performance and applicability of various electron acceptors and mediators used in MFCs. This review also evaluated the corresponding performance, advantages and disadvantages, and future potential applications of select electron acceptors (e.g., nitrate, iron, copper, perchlorate) and mediators.

## Introduction

A microbial fuel cell (MFC) is a bioelectrochemical device that can generate electricity by the use of electrons obtained from the anaerobic oxidation of substrates. Generally, the MFC consists of two parts, an anode and a cathode, which are separated by a proton exchange membrane (PEM). Anaerobic oxidation of organic substances such as acetate, glucose, lactate, ethanol (summarized by Pant et al., 2010) occurs in the anode compartment, during which process protons, electrons and carbon dioxide are released. In this case, the protons and electrons pass through the anode chamber to the cathode chamber via the PEM and an external circuit respectively. This electron transfer from the anode to the cathode produces an electricity current (Logan et al., 2006; Venkata Mohan et al., 2008; Kim and Lee, 2010; Mao et al., 2010; Samrot et al., 2010; Ishii et al., 2013). MFCs can be used for wastewater treatment since organic materials can be easily oxidized as fuel in the anode compartment. In recent years, MFC-based systems have also been used in a number of new applications such as hydrogen production, seawater desalination, biosensors and microbial electro synthesis (Cheng and Logan, 2007; Cao et al., 2009; Rabaey and Rozendal, 2010; Zhang and Angelidaki, 2011).

Despite promising initial results, MFCs have not been able to go further than the pilot scale due to a number of limitations (Liu and Logan, 2004; Donovan et al., 2011). The power output of the MFC depends on several factors such as type of substrate, exoelectrogenic microorganisms, circuit resistance, electrode material, reactor configuration and electron acceptors (Pant et al., 2010; Kim et al., 2011). Different electron acceptors exhibit physically and chemically different properties (e.g., oxidation potential) and therefore affect the efficiency of electricity production. Therefore, investigation of the applicability of new electron acceptors in MFCs has gained importance in recent years as they have a significant impact on electricity generation.

Oxygen is the most common electron acceptor used in the cathode compartment due to its high oxidation potential and the fact that it yields a clean product (water) after reduction. However, most studies show that the oxygen supply to the cathode compartment is energy consuming (Strik et al., 2010). Although the oxygen in the air can be used directly by using an air cathode, contact difficulties in the cathode-air surface and the need for expensive catalysts are the disadvantages of oxygen utilization (Heijne et al., 2007).

The use of alternative electron acceptors may not only increase the power generation and reduce the operating costs, but also expands the application scope of MFCs. It has been recently found that some recalcitrant compounds can be treated in the cathode as an electron acceptor (Gu et al., 2007). These findings suggest that MFCs can be used to control environmental pollutants. For example, nitrate is a well-known pollutant in wastewater streams. Since the redox potentials of nitrate and oxygen are very close to each other, nitrate can be used as an electron acceptor in the cathode compartment (Jia et al., 2008). In this case, the nitrate is reduced to nitrogen gas by the denitrification process in the cathode compartment. Apart from nitrate, some heavy metals such as copper (Tao et al., 2011b), iron and mercury (Wang et al., 2011) can also be used as electron acceptors and thus reduced to less toxic forms. Thus, electricity generation and wastewater treatment take place simultaneously. Recently, Rahimnejad et al. reviewed the effect of anode, cathode, and membrane portions on MFC performance. In addition, the electrode materials used in the anode and cathode compartments are summarized together with some cathodic reactions such as denitrification and iron reduction (Rahimnejad et al., 2015).

Electron acceptors receive electrons from the cathode, and therefore they make a significant contribution to the performance of the MFC. Although there are many studies on different electron acceptors in the literature, no comprehensive review of this field is available. For this reason, the various electron acceptors used in MFCs have been systematically compiled in this paper and evaluated in terms of performance, advantages and application areas in wastewater treatment. Finally, this paper suggests prospects for future development.

## Structure of MFC and oxygen as terminal electron acceptor

**Figure 1:**
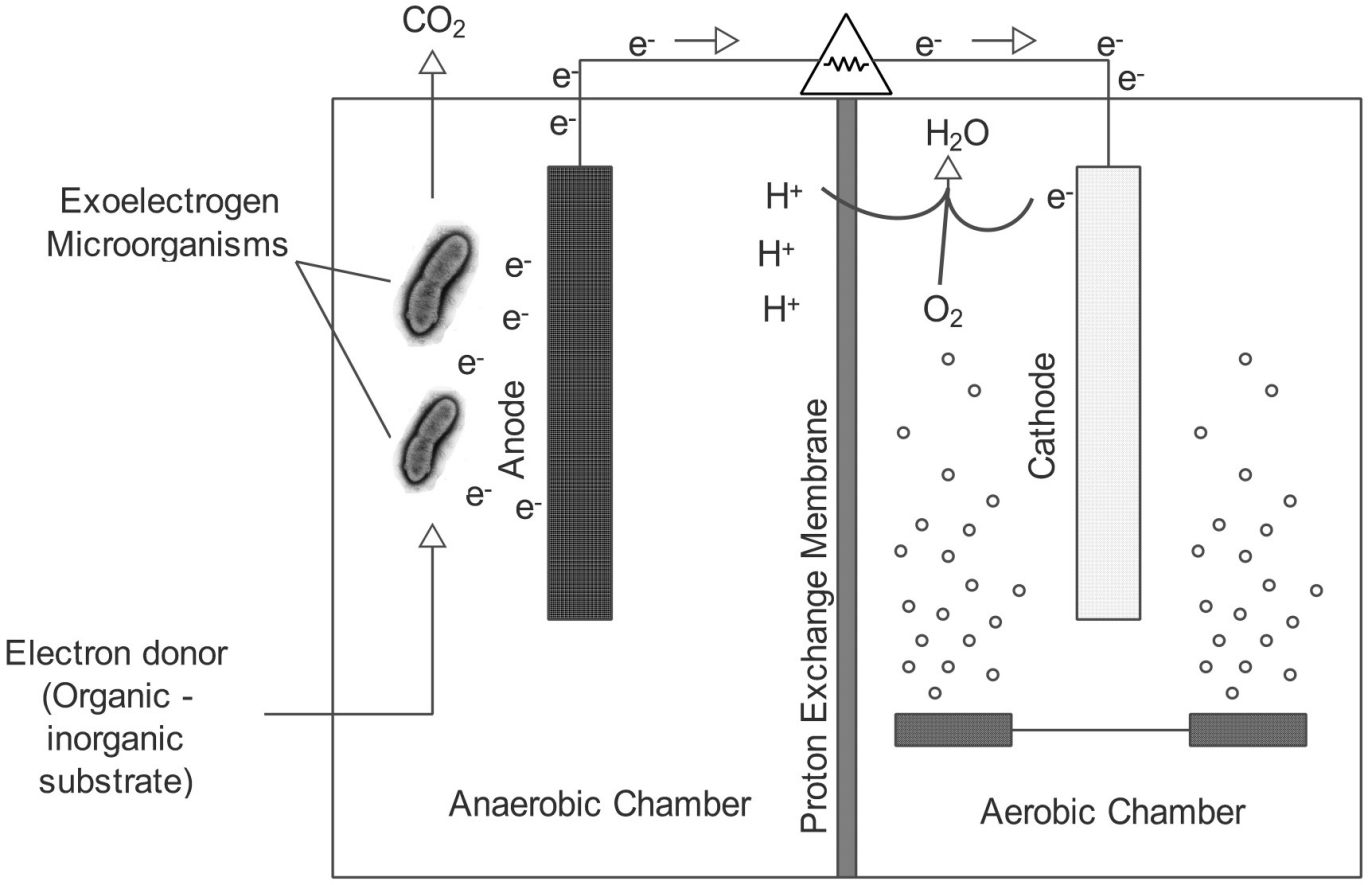
**Schematic representation of a two-chambered MFC**.

A typical MFC consists of two chambers, an anode and a cathode, separated by a PEM membrane (Figure 1). Electrons are transported from the anode compartment to the cathode compartment by external circuit, where they combine with protons and oxygen to form water according to the following reaction

(1) $$\begin{array}{r} {\text{O}}_{2}+4{\text{H}}^{+}+4{\text{e}}^{-}\to 2{\text{H}}_{2}\text{O }\left({\text{E}}^{0}=1.23\text{ V}\right) \end{array}$$

As can be seen in Equation (1), oxygen is continuously consumed to maintain the potential for electricity generation. Oxygen can be provided in the cathode compartment by bubbling the water or by using an air cathode. Oxygen has a higher redox potential than many other electron acceptors, and therefore it is widely regarded as a good cathodic electron receiver (Oh et al., 2004). However, the poor contact of oxygen with the electrode and the slow rate of reduction of the oxygen on the normal carbon electrode are disadvantages that limit the use of oxygen in MFCs (Rhoads et al., 2005). Although the cathodic reaction can be improved by the use of catalytic-coated electrodes, catalysts are often expensive and rare metals (Zhou et al., 2011).

## Alternative electron acceptors

### Ferriciyanide

Besides oxygen, ferricyanide is another common electron donor used in MFC studies since its concentration is not limited to solubility like in the case of oxygen (Rhoads et al., 2005). Although the standard redox potential of ferricyanide (given in Equation 2) is not as high as that of oxygen, it has much lower overpotential, which results in not only a faster reaction rate but also much higher power output (Rabaey et al., 2003; Schröder et al., 2003; Aelterman et al., 2006). It was reported that ferricyanide with the carbon electrode produced 50–80% higher power than oxygen with Pt-carbon cathode due to increased mass transfer efficiencies and larger cathode potential (Oh et al., 2004).

(2) $$\begin{array}{r} {\text{Fe}\left(\text{CN}\right)}_{6}^{3-}+{\text{e}}^{-}\to {\text{Fe}\left(\text{CN}\right)}_{6}^{4-} \end{array}$$

Although ferricyanide is an excellent electron acceptor in terms of power generation, it has been understood that potassium ferricyanide is not practically sustainable. It is toxic, and chemical regeneration/recycling is difficult. For this reason, the use of ferricyanide is limited to basic laboratory studies (Logan et al., 2006). However, ferricyanide is still an important cathodic electron acceptor to prove some important concepts in the laboratory due to its stability and high system performance. For example, Aelterman et al. (2006) conducted performance experiments with MFCs operated in series and in parallel to each other. They used hexacyanoferrate cathode, and six independent continuous MFC units produced the maximum hourly average power output of 258 W/m^3^ in the stacked configuration (Aelterman et al., 2006). Ferricyanide has also been used to compare the performance of electrode materials due to its catalytic activity. In a recent study, three different MFC processes were used to remove nitrogen and carbon from wastewater. The tested MFC types were (1) continuous operation, (2) continuous operation with ferricyanide and (3) continuous operation with oxygen, and the highest current, carbon and nitrogen removal was observed in continuously operating MFC with ferricyanide. The currents obtained are 0.833 and 0.589 V for ferricyanide and oxygen respectively. With ferricyanide, the carbon and nitrogen removals are 36 and 9% higher than that removed with oxygen respectively (Zain et al., 2015).

### Nitrogen species

Nitrate is one of the common types of nitrogen that is widely found in waters, and causes a variety of serious environmental and health problems that threaten human and animal health (Demirel et al., 2014; Sahinkaya et al., 2015). In this respect, nitrate in drinking water is limited to 44.43 mg/L in the US and 50 mg/L in Europe (Shen et al., 2009). The application of biocathots has made nitrate usable as an electron acceptor in MFCs for denitrification and electricity generation. The feasibility of nitrate as a cathodic electron acceptor in MFCs was first demonstrated by Clauwaert et al. (2007). In this study, the denitrification by microorganisms took place in a tubular reactor without an energy input (Clauwaert et al., 2007). In the same period Lefebvre et al. (2008) investigated the same cathodic process in a two-chambered MFC. In their study, 95.7% of nitrate was removed at the cathode using acetate as an anodic substrate, and 73 ± 4% of the total nitrogen was converted to N_2_ gas through electrochemical denitrification according to Equations (3–6). However, only 0.095 V was obtained as the maximum cell potential at external resistance of 1000 Ω, which was much lower than that of oxygen reported previously. This may be due to the fact that nitrate has a relatively low redox potential (0.74 V).

(3) $$\begin{array}{r} {\text{NO}}_{3}^{-}+2{\text{e}}^{-}+2{\text{H}}^{+}\to {\text{NO}}_{2}^{-}+{\text{H}}_{2}\text{O} \end{array}$$

(4) $$\begin{array}{r} {\text{NO}}_{2}^{-}+{\text{e}}^{-}+2{\text{H}}^{+}\to \text{NO}+{\text{H}}_{2}\text{O} \end{array}$$

(5) $$\begin{array}{r} \text{NO}+{\text{e}}^{-}+{\text{H}}^{+}\to {½\text{N}}_{2}\text{O}+{½\text{H}}_{2}\text{O} \end{array}$$

(6) $$\begin{array}{r} {½\text{NO}}_{2}+{\text{e}}^{-}+{\text{H}}^{+}\to {½\text{N}}_{2}+{½\text{H}}_{2}\text{O} \end{array}$$

In order to further investigate the concept and broaden this application, Virdis et al. (2008) demonstrated a novel process which is an integration of MFC and aerobic nitrification technology for simultaneous carbon and nitrogen removal. In this process, the wastewater containing ammonium and organic matter was initially fed to the anode compartment for the oxidation of the organic material and release of the electrons. The effluent from the anode was then fed to an external aerobic nitrification vessel for oxidation of ammonium to nitrate. This nitrate-enriched stream was finally fed to the cathode compartment of the MFC for denitrification where electrons degraded nitrate. The electrons produced at the beginning were transported to nitrate, which was used as an electron acceptor at the end of the process. In this system, which is called loop configuration, a volumetric power density of 34.6 ± 1.1 W/m^3^ and a nitrogen removal rate of up to kg COD/(m^3^ NCC.d) were obtained (NCC: Net cathodic compartment; Virdis et al., 2008). However, this process has its own drawback, which is the low nitrogen removal in the effluent due to the crossover of ammonia from the anode to the cathode through the cation exchange membrane.

To further address this shortcoming, Virdis et al. (2010) integrated the nitrification stage into the cathode chamber where simultaneous nitrification and denitrification (SND) were accomplished. In such a system, denitrification can still occur at a higher dissolved oxygen level than that of a conventional SND process. The main explanation for this finding was the formation of a micro-environment on the porous surface of graphite granule where denitrifying bacteria could grow (Virdis et al., 2010). Studies on simultaneous nitrification-denitrification in MFCs are becoming more successful and various systems are being developed. A combined use of the membrane aerated biofilm process and MFC process was proposed by Yu et al. (2011) for simultaneous nitrification, denitrification and organic carbon removal in a single two-chambered MFC system. In this system, 97 and 52% removal efficiencies for total carbon and nitrogen respectively were obtained. Xie et al. (2011), developed an oxic/anoxic biocathode system for simultaneous carbon and nitrogen removal. The idea behind the rearrangement is to remove ammonium and nitrate in the oxic and anoxic biocathode respectively, while COD is being oxidized in the anode compartment. With this system, the maximum power densities for oxic and anoxic biocathots were 14 and 7.2 W/m^3^ respectively. On the other hand, the maximum COD, ${\text{NH}}_{4}^{+}$ and TN removal rates were 98.8, 94.7, and 97.3% respectively.

Beside the denitrification of nitrate at the cathode, the electrochemical reduction of nitrate at an abiotic cathode has also been explored. Fang et al. (2011) reported that nitrate can be reduced from 49 to 25 mg N/L in the cathode compartment and a power density of up to 7.2 mW/m^2^ can be obtained in this process at 470 Ω resistance (Fang et al., 2011). The reduction products of nitrate were mainly ammonia (51.8%) and trace amounts of nitrite (0.6%).

While nitrate reduction at the cathode has been extensively studied, nitrite as an important intermediate product from nitrate reduction has received little attention so far. Virdis et al. (2008) suggested that nitrite as an efficient terminal electron acceptor at the cathode of a MFC could reduce the carbon to nitrogen ratio (Virdis et al., 2008). Puig et al. (2011) demonstrated that nitrate and nitrite can be used interchangeably as an electron acceptor by exoelectrogenic bacteria for nitrogen reduction. However, nitrite is oxidized in the presence of oxygen by biological or electrochemical processes at the cathode, which affects the electricity production (Puig et al., 2011).

Up-to-date studies on MFC for nitrate removal can include field applications. Organic pollutants in river X in Romania were used for electricity generation and the nitrate in the same river was used as an electron receiver. A power density of 88 mW/m^2^ was achieved at a current density of 310 mA/m^2^ in a single compartment MFC. Organic pollution and nitrate removal efficiencies were 97 and 96%, respectively (Cucu et al., 2016).

Nitrous oxide is an important intermediate between the steps of the denitrification process shown in Equations (3–6). Reducing N_2_O emissions is an urgent issue as it is an important greenhouse gas. According to the thermodynamic principle, N_2_O has the potential to be a more suitable electron acceptor compared to the other oxidized nitrogen intermediates in the denitrification pathway. In a study conducted by Desloover et al. (2011), N_2_O removal rates ranging from 0.76 to 1.83 kg N/m^3^ NCC were obtained at the cathode chamber.

### Persulfate

Persulfate is used in many applications such as clarifying swimming pools, hair bleaching, micro-etching of copper printed circuit boards, total organic carbon analysis and destructing soil and groundwater contaminants. It is considered to be hazardous waste because it is an oxidizing agent (Li J. et al., 2009). Applicability of persulfate in MFCs is possible with its standard oxidation reduction potential of 2.12 V, which is higher than many electron acceptors (e.g., permanganate) used in MFCs. When persulfate is used as the electron acceptor, 1 mole S_2_${\text{O}}_{8}^{2+}$ receives 2 electrons and forms ${\text{SO}}_{4}^{2-}$ (Equation 7).

(7) $$\begin{array}{r} {\text{S}}_{2}{\text{O}}_{8}^{2-}+2{\text{e}}^{-}\to 2{\text{SO}}_{4}^{2-} \end{array}$$

Because of the above properties, persulfate was used as electron acceptor. It was found that power density was doubled when K_3_Fe (CN)_6_ was replaced with persulfate in MFC (166.7 vs. 83.9 mW/m^2^). One drawback of MFC with K_2_S_2_${\text{O}}_{8}^{2+}$ could be the lower cell performance than MFC with K_3_Fe(CN)_6_ at medium to high current densities. This case was explained by the faster electron reduction kinetics of ferricyanide solution on the surface of the carbon electrode (Li J. et al., 2009).

### Permanganate

Under both acidic and alkaline conditions, permanganate is reduced to manganese dioxide by receiving three electrons as shown in the Equations (8, 9). This property of permanganate makes it a potential electron acceptor. In acidic conditions, permanganate is expected to show higher power output since its oxidation potential is higher than it is in alkaline conditions. Therefore, studies in different pH values were done to investigate the performance of permanganate in MFCs (You et al., 2006).

(8) $$\begin{array}{r} {\text{MnO}}_{4}^{-}+4{\text{H}}^{+}+3{\text{e}}^{-}\to {\text{MnO}}_{2}+2{\text{H}}_{2}\text{O} \end{array}$$

(9) $$\begin{array}{r} {\text{MnO}}_{4}^{-}+2{\text{H}}_{2}\text{O}+3{\text{e}}^{-}\to {\text{MnO}}_{2}+4{\text{OH}}^{-} \end{array}$$

In a previous study, a power density of 115.60 mW/m^2^ at a current density of 0.017 mA/cm^2^ was observed by using permanganate as an electron acceptor, which was 4.5 and 11.3 fold higher than that produced from hexachnoferrate (25.62 mW/m^2^) and oxygen (10.2 mW/m^2^) respectively. Moreover, in the same study, a bushing MFC using permanganate as the electron acceptor achieved the maximum power density of 3986.72 mWm^2^ at 0.59 mA/cm^2^. Therefore, it is worth pointing out that permanganate can be an efficient cathodic electron acceptor in MFCs (You et al., 2006).

However, there are also some drawbacks existing in this application. For example, like other soluble electron acceptors, depletion of permanganate during electricity generation requires continuous liquid replacements. Moreover, since the cathode potential is mainly dependent on the solution pH, pH control is required for stable power output, which may only be applied to small-scale power supplies as suggested by the authors. On the other hand, the advantage of this system is that it does not require catalysis (You et al., 2006). In a more recent study, the best permanganate concentration was studied in terms of electricity production. The maximum power density with 400 mM of potassium permanganate and the current density at this power density were found to be 93.13 mW/m^2^ and 0.03 mA/cm^2^ respectively (Eliato et al., 2016).

### Manganese dioxide

Studies have reported that manganese dioxide is a good cathode material and catalysis for battery and alkalinine fuel cells (Li et al., 2010). MnO_2_/Mn^2+^ redox couple can be used to transfer electrons from the cathode to an electron acceptor. Rather than direct utilization of oxygen, the use of electron mediators between cathode and oxygen is more efficient because of the difficulties in the direct utilization of oxygen (i.e., low solubility). The possibility of biomineralized manganese oxides was investigated by Rhoads et al. (2005). The reaction begins with the accumulation of manganese dioxide on the cathode surface and subsequent reduction with electrons from the anode. The reaction results in the release of manganese ions which are subsequently reoxidized to manganese dioxide by manganese-oxidizing bacteria *(Lepthothrix discophora* SP-6), and the cycle continues (Equations 10–12). Maximum power density of 126.7 ± 31.5 mW/m^2^ was obtained (with 50 Ω resistor) from above redox cycle (Rhoads et al., 2005).

(10) $$\begin{array}{r} {\text{MnO}}_{2}+{\text{H}}^{+}+{\text{e}}^{-}\to \text{MnOO}{\text{H}}_{\left(\text{S}\right)} \end{array}$$

(11) $$\begin{array}{r} \text{MnOOH}+3{\text{H}}^{+}+{\text{e}}^{-}\to {\text{Mn}}^{2+}+2{\text{H}}_{2}\text{O} \end{array}$$

(12) $$\begin{array}{r} {\text{Mn}}^{2+}+{\text{O}}_{2}+2{\text{e}}^{-}\to {\text{MnO}}_{2} \end{array}$$

Manganese dioxide can be used not only in the electron mediator mechanism but also as an alternative cathode catalyst to platinum due to its low cost (Liew et al., 2015). Using manganese dioxide as an alternative catalyst, the maximum volumetric anode density of 3,773 ± 347 mW/m^3^ was obtained with a tube MFC. It could be, therefore, noted that using MnO_2_ instead of Pt could serve as a suitable option for real applications due to its low cost (Zhang L. et al., 2009).

### Mercury (Hg)

Since the redox potential of mercury, which is about −320 mV (Hg^2+^), is higher than that of NADH/NAD^+^, it can be accepted as an alternative electron acceptor (Wang et al., 2011). By using mercury in MFC, its removal from the aquatic environment can be achieved simultaneously with electricity production. The possible removal mechanism is to precipitate Hg^2+^ in the presence of Cl^−^ as shown in Equation (13), and subsequent reduction by the electrons at the cathode (Equation 14). Maximum power density of 433.1 mW/m^2^ was obtained from the above process, while the end products were elemental Hg in the cathode surface and Hg_2_CI_2_ as deposits on the bottom of the cathode chamber (Wang et al., 2011).

(13) $$\begin{array}{r} 2{\text{Hg}}^{2+}+2{\text{Cl}}^{-}\to {\text{Hg}}_{2}{\text{Cl}}_{2}\left(\text{s}\right) \end{array}$$

(14) $$\begin{array}{r} {\text{Hg}}_{2}{\text{Cl}}_{2}\left(\text{s}\right)+2{\text{e}}^{-}\to 2\text{Hg}\left(\text{I}\right)+2{\text{Cl}}^{-} \end{array}$$

### Iron (Fe)

Iron can be used as an electron mediator to enhance the performance in the cathode compartment. The most common redox couple used in MFCs is Fe^+3^/Fe^+2^. Ferric iron can be reduced to ferrous iron in the cathode chamber according to Equation (15).

(15) $$\begin{array}{r} {\text{Fe}}^{3+}+{\text{e}}^{-}\to {\text{Fe}}^{2+} \end{array}$$

This reversible electron transfer reaction provides several advantages such as fast reaction, high standard potentials, biological degradability (Heijne et al., 2006) and release of some valuable compounds such as Phosphate (Fischer et al., 2011). In a study where this redox was coupled together with a bipolar membrane and graphite electrode combination, the maximum power density of 0.86 W/m^2^ at a current density of 4.5 A/m^2^ was obtained (coulombic efficiency and energy recovery were 80–95 and 18–29% respectively; Heijne et al., 2006). In order to complete the Fe^3+^/Fe^2+^ redox cycle, an oxidative mechanism is needed. To achieve this, Heijne et al., used an acidophilic chemolithoautotrophic microorganism—*Acidithiobacillus ferrooxidans* to oxidize ferrous iron and investigated the performance of the MFC with continuous ferrous iron oxidation (Heijne et al., 2007). Oxidation of ferrous iron to ferric iron resulted in a 38% higher power output (1.2 W/m^2^ and a current of 4.4 A/m^2^) than that which was obtained in their previous study.

**Figure 2:**
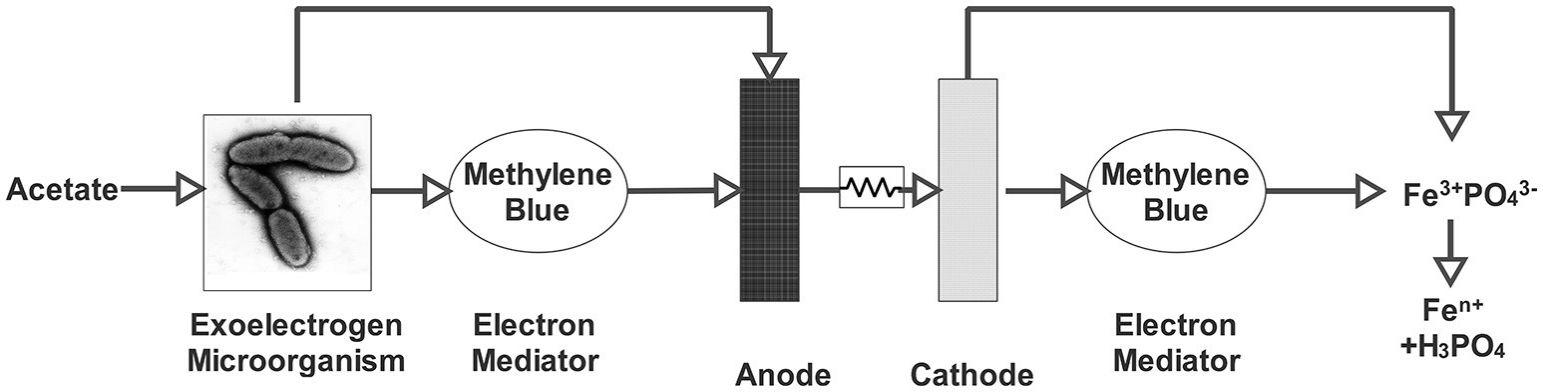
**MFC for the mobilization of orthophosphate from FePO_**4**_ (Fischer et al., **2011**)**.

Besides being an electron mediator, iron can also be used as an electron acceptor. In another study, iron in the form of FePO_4_ was used (Figure 2). FePO_4_ is a compound found in sewage sludge, which not only has a potential as an electron acceptor due to its Fe^3+^ content, but also has a great importance due to its orthophosphate content (Equation 16; Fischer et al., 2011).

(16) $$\begin{array}{r} 3{\text{e}}^{-}+3{\text{H}}^{+}+{\text{Fe}}^{3+}{\text{PO}}_{4}↔{\text{H}}_{3}\text{PO},{\text{H}}_{2}{\text{PO}}_{4}^{-},{\text{HPO}}_{4}^{2-},{\text{PO}}_{4}^{3-} \end{array}$$

Phosphorus is an essential element for both agricultural and industrial production. This important element, however, is assumed to be depleted within 50–100 years (Cordell et al., 2009). Since it is also one of the primary causes of eutrophication, it is essential to consider the recovery of phosphate rather than its disposal (Usharani and Lakshmanaperumalsamy, 2010).

The MFC could reduce FePO_4_ by delivering necessary electrons and protons. When iron cations are reduced by the electrons, iron and phosphate are separated and mobilized orthophosphate (${\text{PO}}_{4}^{3-}$) is released into the solution. Released orthophosphate can be further precipitated as struvite (NH_4_MgPO_4_) by adding stoichiometric amounts of Mg^2+^ and ${\text{NH}}_{4}^{+}$. By this method, 82% orthophosphate recovery was achieved with a varied current density of 0.1 and 0.7 mA (Fischer et al., 2011).

Compared to other electron acceptors and mediators, ferric iron provided relatively high power densities (Table 1). However, MFC with ferric iron requires a bipolar membrane instead of a cation exchange membrane (CEM). CEMs are not suitable for pH adjustment in the cathode chamber since they carry other cations together with protons. Therefore, either a bipolar membrane or acid addition is required when ferric iron is used (Heijne et al., 2006).

**Table 1:** **Cathodic electron acceptors and the maximum power densities**.

**Type of substrate**	**Type of cathodic electron acceptor**	**Maximum power density**	**References**
Acetate	Hg^2+^	433.1 mW/m^2^	Wang et al., 2011
Potassium acetate	Ferric iron	0.86 W/m^2^	Heijne et al., 2006
Potassium acetate	Ferric iron	1.2 W/m^2^	Heijne et al., 2007
Glucose	Biologically mineralized manganese-oxides	126.7 ± 31.5 mW/m^2^	Rhoads et al., 2005
Glucose	permanganate	115.60 mW/m^2^	You et al., 2006
Glucose	hexachnoferrate	25.62 mW/m^2^	You et al., 2006
Glucose and sodium acetate	FePO_4_	–	Fischer et al., 2011
Acetate	Potassium persulfate	83.9 mW/m^2^	Li J. et al., 2009
Acetate	Potassium ferricyanide	166.7 mW/m^2^	Li J. et al., 2009
Domestic wastewater	Nitrate	9.7 mW/m^2^	Lefebvre et al., 2008
Sodium acetate	Nitrate	–	Lefebvre et al., 2008
Domestic wastewater	Nitrate	117.7 mW/m^2^	Fang et al., 2011
Sodium acetate	Nitrate	8.15 ± 0.02 W/m^3^	Virdis et al., 2010
Glucose	Ammonium	14 W/m^3^	Xie et al., 2011
Glucose	Nitrate	7.2 W/m^3^	Xie et al., 2011
Acetate	Nitrate	34.6 ±1.1 W/m^3^	Virdis et al., 2008
Glucose	Cu(II) sulfate	314 mW/m^3^	Tao et al., 2011a
Sodium acetate	Cr(IV)	1,600 mW/m^2^	Li et al., 2008
Acetate	Cr(IV)	–	Li Y. et al., 2009
Acetate	Cr(IV)	150 mW/m^2^	Wang et al., 2008
Acetic acid	Triiodide (I_3_)	484.0 mW/m^2^	Li J. et al., 2010
Glucose	H_2_O_2_	22 mW/m^2^	Tartakovsky and Guiot, 2006
Fatty acids and alcohols	CO_2_	–	Villano et al., 2010
Sodium acetate	CO_2_	750 mW/m^2^	Cao et al., 2009
Acetate	ClO_4_	–	Butler et al., 2010
Sulfide and glucose	Vanadium (V)	572.4 ± 18.2 mW/m^2^	Zhang B. et al., 2009
Glucose	Vanadium (V)	614.1 mW/m^2^	Zhang et al., 2010
Acetate	Uranium (IV)	10 mW/m^2^	Williams et al., 2010
Externally supplied voltage	Chlorinated aliphatic hydrocarbons	–	Aulenta et al., 2009
Acetate/Externally supplied voltage	2-chlorophenol	–	Strycharz et al., 2010

The main advantage of this process is that the phosphate is obtained in pure form. Thus, phosphate can be separated from iron and other toxic materials such as As, Pb, Cr. However, low pH is required to keep ferric iron soluble since ferric iron is tent to be precipitated as ferric iron hydroxides at pH values higher than 2.5. These precipitates are reported to be harmful to membrane use. Additionally, in order to shuttle electrons and protons to the Fe^3+^, a cathodic mediator such as methylene blue needs to be supplied, which may hinder its wide application (Fischer et al., 2011). While iron was used as an electron acceptor, up-to-date studies show that it can also be used to prepare efficient catalysts (Nguyen et al., 2016; Santoro et al., 2016).

### Copper

Copper is one of the widespread heavy metals in the soil and aquatic environment, which are mainly emitted from mining and metallurgical industries. Trace amounts of copper is an essential micronutrient to all plants and animals, but high levels of copper can become toxic to all life forms (Alaoui-Sossé et al., 2004). Therefore, the removal of copper is of great importance. Simultaneous copper recovery and energy production in a two-compartment MFC were investigated (Heijne et al., 2010; Tao et al., 2011b). The copper reduction in its basic form is shown in Equation (17).

(17) $$\begin{array}{r} 4{\text{Cu}}^{2+}+8{\text{e}}^{-}\to 4\text{Cu}\left(\text{s}\right) \end{array}$$

Copper recovery in MFC was done by Heijne et al. (2010) by using a bipolar membrane as a pH separator. The maximum power density was 0.80 W/m^2^ at a current density of 3.2 A/m^2^ and over 99.88% removal efficiency was achieved. Pure copper crystals were observed as the main products formed on the cathode surface and no CuO or Cu_2_O was detected. As noted in the previous work of the authors, the bipolar membrane provided low pH in the cathode compartment.

Tao et al. investigated Cu^2+^ reduction in an MFC using a PEM and cupric sulfate solution as catholyte (Tao et al., 2011b). The maximum power density at the initial copper concentration of 6412.5 ± 26.7 mg Cu^2+^/L in glucose-fed MFC was measured as 339 mW/m^3^. High copper removal efficiency (>99%) was obtained when the initial copper concentration was 196.2 ± 0.4 mg Cu^2+^/L and 15 Ω external resistance. In order to further lower the construction cost for this process, Tao et al. (2011a) developed a lab-scale membrane free buffled MFC. At an initial copper concentration of 500 mg/L, a removal efficiency of 70% was observed over a period of 144 h (Tao et al., 2011a). Copper is an attractive electron receiver that can compete with oxygen (Tao et al., 2011b). For this reason, the cathodic copper reduction has broadened the field of MFC applications.

Copper reduction and electricity generation may vary depending on the architectural structure and operational parameters of the reactors. In an up-to-date study, electricity production was investigated by multiple batch cycle operations with different cathode materials (Wu D. et al., 2016). For the copper removal, a carbon rod, a titanium sheet, and stainless steel woven mesh materials were tested as cathode material. Stainless steel woven mesh was found as the most effective and cheap cathode material. When copper reduction is desired in MFC, the deposition of copper on the cathode has a great effect on power density and copper removal.

However, this technology is still in an early stage of development, more developments such as cost-effective reactor design and study of the catalytic behavior of copper for oxygen reduction at the cathode are required. Studies on copper removal in MFC indicate that the power density can be up to 33.6 W/m^3^ depending on parameters such as reactor type, electron source, anode and cathode materials (Wu D. et al., 2016).

### Chromium

The use of chromium as an electron acceptor has been demonstrated in several studies (Li et al., 2008; Li Y. et al., 2009; Sahinkaya et al., 2016). Real and synthetic wastewaters containing chromium were treated in MFCs and chromium reduction and electricity production were accomplished simultaneously. In acidic conditions Cr(IV) can be reduced to Cr(III) by the transfer of six electrons as illustrated in Equation (18).

(18) $$\begin{array}{r} {\text{Cr}}_{2}{\text{O}}_{7}^{2-}+14{\text{H}}^{+}+6{\text{e}}^{-}\to 2{\text{Cr}}^{3+}+7{\text{H}}_{2}\text{O} \end{array}$$

This reduction reaction is thermodynamically feasible with a redox potential of 1.33 V. In a study, with a synthetic wastewater containing 200 mg Cr(IV)/L, the maximum power density of 150 mW/m^2^ was obtained (0.04 mA/cm^2^) and the maximum open circuit voltage was reported as 0.91 V. In this study, low pH was found to have a positive effect on Cr(VI) reduction (Wang et al., 2008). In another study, Li et al., investigated the same process with real electroplating wastewater containing Cr(VI) (Li et al., 2008). In this study, Cr(VI) removal was found to be influenced by the electrode material. Graphite paper and graphite plates were used as cathode material in chrome removal and graphite paper gave better results than graphite plate (power density: 1,600 and 99.5% chromium removal rate for electroplating wastewater containing 204 mg Cr(VI)/L).

Different from conventional Cr(VI) reduction in MFCs, Li Y. et al. (2009) studied the Cr(VI) reduction in an MFC photoelectrochemical cell coupled system. Under light irradiation, 97% Cr(IV) removal was achieved within 26 h at the initial concentration of 26 mg/L (Li Y. et al., 2009). The maximum potential generated under light irradiation and dark controls were 0.80 and 0.55 V, respectively. The authors used rutile coated cathode for waste treatment and solar energy conversion in a single unit of MFC. These synergies between a biocatalyzed anode and a rutile coated cathode promoted the power output and Cr(IV) reduction (Li Y. et al., 2009).

In an up-to-date study, the microbial concentration was increased to improve chromium reduction performance in the cathode chamber. For this, the exoelectrogenic biofilm was enriched in the anode compartment and the system was subsequently established using the anode as biocathode. This new method has increased Cr(VI) reduction efficiency by 2.9 times compared to common biocathots (Wu et al., 2015). Other recent studies on Cr(VI) reduction were focused on self-assembled graphene biocathode applications (Song et al., 2016) and on electrode material modification (Wu X. et al., 2016). Current studies are usually focused on the cathode material for chromium removal. Cr(VI) removal was studied in MFC operated with an alumina (AA)/nickel (Ni) nanoparticles (NPs)-dispersed carbon nanofiber electrode. With the developed electrode, a power density of 1,540 mW/m^2^ was achieved together with the complete reduction of 100 mg Cr(VI)/L at a reduction rate of 2.12 g/(m^3^ h). The columbic efficiency was 93% (Gupta et al., 2017). In another study with abiotic cathode, a power density of 21.4 mW/m^2^ was obtained in the treatment of alkaline Cr(VI) wastewater, while 10 mg/L chromium reduction was achieved within 45 h (Xafenias et al., 2015).

### Triiodide

**Figure 3:**
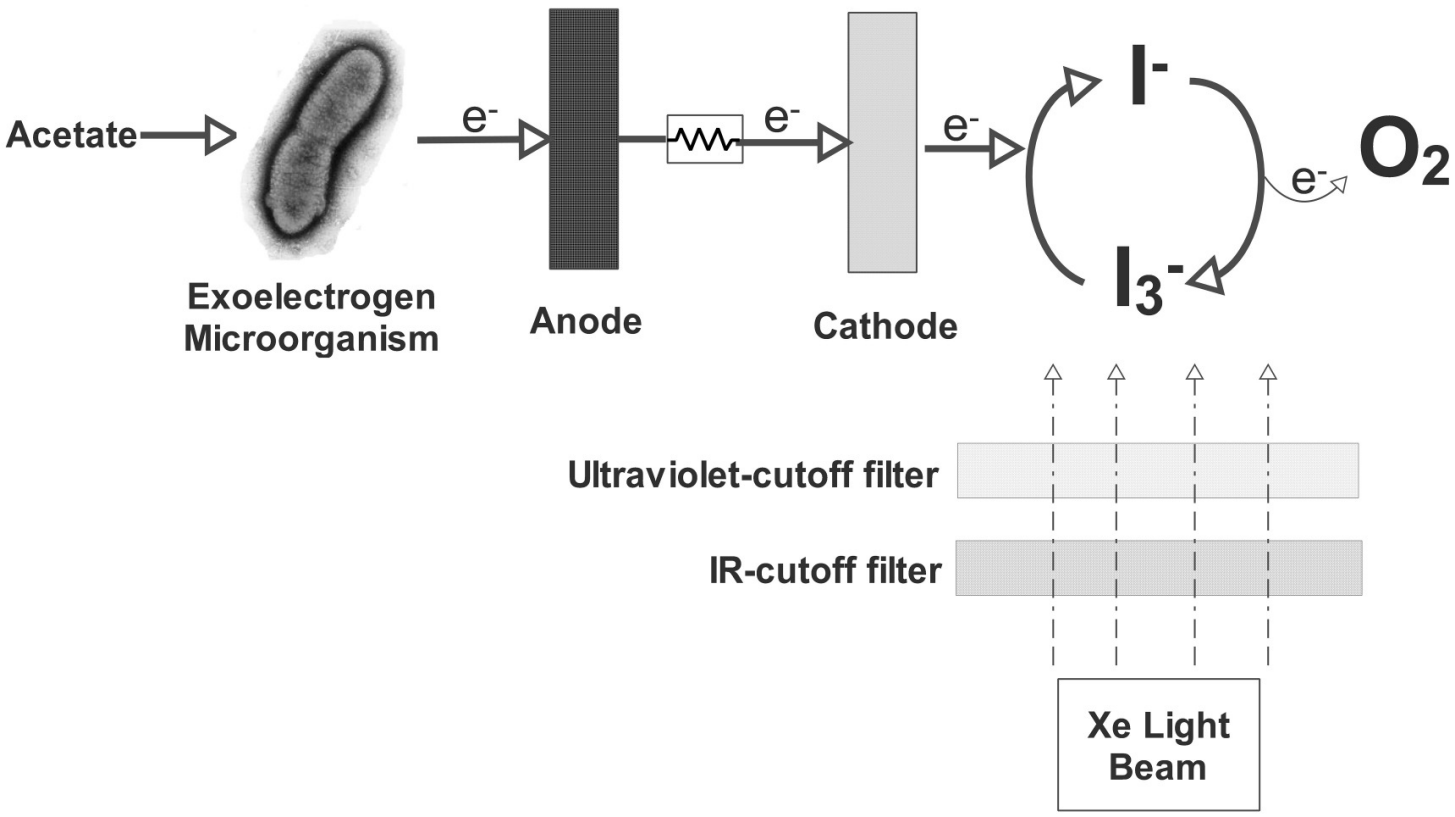
**Schematic view of the MFC using aqueous iodide ion solution as the catholyte (Li et al., **2010**)**.

Similar to Fe^+3^/Fe^+2^ discussed in Section Copper, iodide/iodine redox couple is another redox couple that can serve as the electron mediator (Equation 19). There are some advantages of using this redox couple in the cathode. Since it can be regenerated in the catholyte, there is no depletion of triiodide (${\text{I}}_{3}^{-}$). This regeneration could be done by a photo-driven reaction between I^−^ and oxygen (Equation 20) presented in Figure 3. After the formation of iodine, the combination of iodide anion with an iodine molecule in water forms triiodide (${\text{I}}_{3}^{-}$) and the cycle continues (Equation 21). It also demonstrates the feasibility of using carbonaceous materials as the cathode. Triiodide is stable at both acidic and alkaline conditions. Because of all these properties, iodide/iodine redox couple could easily apply as an electron mediator in the cathode chamber for accepting and transferring electrons. The feasibility of this redox couple as the electron acceptor or mediator was first demonstrated using a two-chambered MFC (Li J. et al., 2010). Maximum power density of 484.0 mW/m^2^ was obtained with 1.2 mM ${\text{I}}_{3}^{-}$ and 0.2 M KI. (Li J. et al., 2010).

(19) $$\begin{array}{r} {\text{I}}_{3}^{-}+2{\text{e}}^{-}\to 3{\text{I}}^{-} \end{array}$$

(20) $$\begin{array}{r} 4{\text{I}}^{-}+{\text{O}}_{2}+4{\text{H}}^{+}+h\upsilon \to 2{\text{I}}_{2}+2{\text{H}}_{2}\text{O} \end{array}$$

(21) $$\begin{array}{r} {\text{I}}_{2}+{\text{I}}^{-}\to {\text{I}}_{3}^{-} \end{array}$$

The present investigations are mainly carried out with H type MFCs, more efficient reactor design for high power generation is still required. It should be noted that ${\text{I}}_{3}^{-}$ ion is toxic to electrochemically active microbes in the anodic chamber. Therefore, this negative effect should be taken into consideration when designing a new configuration for better performance (Li J. et al., 2010).

### Hydrogen peroxide

Because of its strong oxidizing properties, H_2_O_2_ is used as an electron acceptor and its mechanism is presented in the following equation.

(22) $$\begin{array}{r} {\text{H}}_{2}{\text{O}}_{2}\left(\text{aq}\right)+2{\text{H}}^{+}+2{\text{e}}^{-}\to 2{\text{H}}_{2}\text{O}\left(\text{I}\right) \end{array}$$

The oxygen concentration used in the cathode section can also be added to hydrogen peroxide. The use of hydrogen peroxide has been reported to provide stability in long-run operations in MFC (Tartakovsky and Guiot, 2006).

In a comparison of air with hydrogen peroxide, the power density in the air-operated MFC was 7.2 mW/m^2^ while it increased to 22 mW/m^2^ with hydrogen peroxide. Liquid hydrogen peroxide provides high levels of oxygen. This ensures high performance in long-run operation (Tartakovsky and Guiot, 2006).

While H_2_O_2_ is used to remove contaminants with hydroxyl radicals formed as a result of reaction with fenton, the remaining H_2_O_2_ must be removed. For this purpose, Zhang et al. have developed an innovative bioelectro-fenton system that uses an alternative switching to operate the system in microbial electrolysis cell (MEC) or MFC mode. In the MEC mode, methylene blue was removed with H_2_O_2_, while the residual H_2_O_2_ was removed in the cathode as an electron acceptor. In this system, 50 mg/L of methylene blue was removed in the MEC system while 180 mg/L of residue H_2_O_2_ was used as an electron acceptor in MFC to produce a maximum current density of 0.49 A/m^2^. With the study, H_2_O_2_ was effectively controlled and contaminant removal was ensured (Zhang Y. et al., 2015).

### Carbon dioxide

Thermodynamically, CO_2_ reduction has a very low redox potential and its use in the cathode compartment produces a very low voltage. The CO_2_ reduction potential is −0.420 V at neutral pH. However, the cathode potential must be higher than the anode potential in order to generate electricity. For this reason, external energy must be supplied in order to provide CO_2_ reduction (Cao et al., 2009).

Cao et al. (2009) demonstrated the CO_2_ reduction driven by sunlight with a biocathode MFC. Electrons could be received by the carbon dioxide, according to the following equation (Equation 23), where (CH_2_O) represents the biomass. In this way, CO2 reduction is provided together with biomass production. This bio-reaction allows the CO_2_ sequestration.

(23) $$\begin{array}{r} {\text{HCO}}_{3}^{-}+4{\text{e}}^{-}+5{\text{H}}^{+}\to {\text{CH}}_{2}\text{O}+2{\text{H}}_{2}\text{O} \end{array}$$

Another application of CO_2_ in the cathodic chambers is to reduce carbon dioxide to methane (Equation 24; Villano et al., 2010). Since both electrons and CO_2_ are released during the oxidation of organic matter, these substances may participate in the production of methane. Villano et al. (2010) first demonstrated the feasibility of this concept using a two-chambered MFC. This process has some advantages. Firstly, the methanogens are protected from possible inhibitors present in the wastewater by separating the oxidation part of the organic matter from the methane production. Secondly, this process consumes less energy because there is no need to heat the cathode section to maintain the temperature. In addition, this process leads the operation of anaerobic digestion and methane producing steps in the series. Therefore, the system is also effective at low substrate concentrations (Villano et al., 2010).

(24) $$\begin{array}{r} {\text{CO}}_{2}+8{\text{H}}^{+}+8{\text{e}}^{-}\to {\text{CH}}_{4}+2{\text{H}}_{2}\text{O} \end{array}$$

In recent years, reduction of CO_2_ to biofuels or commodity chemicals in the cathode with the help of microbes and externally supplied electricity has gained tremendous attention. Researches in this area have opened a new door for biofuel or chemical production by overcoming the limitation in natural photosynthesis processes and corresponding processes have been commonly named as microbial electrosynthesis or recently as artificial photosynthesis. Via these processes, multicarbon compounds such as acetate (Patil et al., 2015), acetic acid (Gildemyn et al., 2015), butyrate (Ganigué et al., 2015) and ethanol (Pant et al., 2010) could be produced as a form of energy storage.

### Perchlorate

Perchlorate is a drinking water contamination of interest due to its high mobility and inhibitory effect on thyroid functions (Cetin et al., 2015; Ucar et al., 2017). Among the treatment alternatives, biological reduction is a cost effective method (Ucar et al., 2015a,b, 2016). Reduction of ${\text{CIO}}_{4}^{-}$ is shown as illustrated in Equation (25) (Ye et al., 2012).

(25) $$\begin{array}{r} {\text{CIO}}_{4}^{-}+8{\text{H}}^{+}+8{\text{e}}^{-}\to {\text{CI}}^{-}+4{\text{H}}_{2}\text{O} \end{array}$$

Butler et al. (2010) reported the reduction of perchlorate to chloride by using highly active perchlorate reducing microbial community in the cathode chamber (Butler et al., 2010). In a MFC operated using acetate and perchlorate, 0.28 mA average current was obtained. The maximum perchlorate removal rate at this point was 24 mg/(L^.^d).

With this method, it is possible to purify perchlorate, which can be found in ground waters. However, in sediment waters, perchlorate is usually found in μ/L range, and electricity production with such low concentrations can be difficult. Nitrate is the most common pollutant found in groundwater. Thus, perchlorate and nitrate removal can be considered together in MFC.

In a recent study conducted for this purpose, nitrate and perchlorate removal were investigated in the autotrophic denitrification biocathode. In acetate-fed MFC, 87.05 and 53.14% of the influent nitrate and perchlorate were removed respectively. The optimum ${\text{NO}}_{3}^{-}$/${\text{CIO}}_{4}^{-}$ ratio is reported as 1:1 (Jiang et al., 2017). Acetate has been reported as the most suitable electron source for perchlorate and nitrate reductions (Lian et al., 2017). Reduction of nitrate and perchlorate with acetate can be carried out at high efficiency, but in the case of nitrates in drinking water, the use of organic electron sources such as acetate is likely to lead to unused acetate in the effluent. In such cases, the use of inorganic electron sources such as sulfur may be more appropriate. Inorganic electron donors also have their own disadvantages, for example, when sulfur is used, sulfate and acidity can occur in the effluent. However, in recent studies, the use of the advantages of both systems in the removal of nitrate and perchlorate from drinking and underground waters and the elimination of disadvantages are becoming increasingly widespread (Ucar et al., 2015b, 2017).

### Vanadium

Another example of the use of MFC in pollutant removal is vanadium removal. Vanadium is usually found in wastewaters of vanadium mines and pentoxide processing activities (Carpentier et al., 2003). Vanadium has high redox potential in acidic conditions and can be successfully used in MFC (Zhang B. et al., 2009). At the vanadium reduction, both organic and inorganic compounds can be used as electron donors (Zhang et al., 2010). Zhang B. et al. (2009) used sulfide and glucose as the electron source to reduce vanadium (Equations 26–30).

The authors demonstrated the removal of sulfide and vanadium in the anode and cathode chambers of MFC respectively (Equations 26–30). Sulfur and vanadium removal rates were 84.7 ± 2.8 and 25.3 ± 1.1%, respectively, with the maximum power density of 572.4 ± 18.2 mW/m^2^.

Anode chamber

(26) $$\begin{array}{r} {\text{HS}}^{-}\to {\text{S}}^{0}+{\text{H}}^{+}+2{\text{e}}^{-} \end{array}$$

(27) $$\begin{array}{r} {\text{S}}^{0}+4{\text{H}}_{2}\text{O}\to {\text{SO}}_{4}^{2-}+8{\text{H}}^{+}+6{\text{e}}^{-} \end{array}$$

(28) $$\begin{array}{r} {\text{C}}_{6}{\text{H}}_{12}{\text{O}}_{6}+6{\text{H}}_{2}\text{O}\to 6{\text{CO}}_{2}+24{\text{H}}^{+}+24{\text{e}}^{-} \end{array}$$

Cathode Chamber

(29) $$\begin{array}{r} {\text{VO}}_{2}^{+}+2{\text{H}}^{+}+{\text{e}}^{-}\to {\text{VO}}_{2}^{2+}+{\text{H}}_{2}\text{O} \end{array}$$

(30) $$\begin{array}{r} 6{\text{O}}_{2}+24{\text{H}}^{+}+24{\text{e}}^{-}\to 12{\text{H}}_{2}\text{O} \end{array}$$

Zhang et al. (2010) further studied the factors affecting the removal of sulfide and Vanadium (V) in MFCs respectively. It has been reported that the initial sulfur concentration has an effect on microbial activity (Zhang et al., 2010). As the initial sulfide concentration increased, microbes in the anode compartment became less effective, which resulted in a long lag time and decreased sulfide removal efficiency from 95.2% (50 mg/L) to 47.5% (200 mg/L) while average V(V) removal was 23.7 ± 4.7% in terms of V(IV) formation (with an initial V(V) concentration of 500 mg/L).

Anodic electrolyte conductivity is another factor affecting vanadium (V) removal and electricity production (Zhang et al., 2010). Increased anode electrolyte conductivity considerably raised the sulfur and vanadium reduction rates. This can be explained by the increased electron transfer rate at enhanced conductivity. Increasing anode electrolyte conductivity to 12.3 mS/cm increased V(IV) generation up to 36.0 ± 1.6%. The initial concentration of Vanadium (V) is also the factor affecting the system performance. When the initial V(V) concentration is increased, the rate of V(IV) formation is also increased. The authors noted that the optimum initial V(V) concentration for 100 mg/L sulfide was 500 mg/L. Further increases in V(V) concentration resulted in a saturation in V(IV) generation. In addition, Vanadium (V) removal rate increased with the decrease of pH. Acidic conditions were necessary to compensate for the slower proton transport rate through the membrane. Under optimized conditions, average removal rates of sulfide and V(V) were 82.2 and 26.1% respectively, while the maximum power density was 614.1 mW/m^2^ (Zhang et al., 2010).

Similar power densities were found in more recent studies. Hao et al. obtained a power density of 543.4 mW/m^2^ at the end of 12 h of operation with a vanadium reduction of 93.6% (Hao et al., 2015). In another study, V was simultaneously reduced in both anode and cathode compartments. Power density of 419 ± 11 mW/m^2^ was achieved while initial vanadium concentrations in the anode and cathode were 75 and 150 mg/L respectively. The total vanadium removal rate was reported as 76.8 ± 2.9 while the final reduction product was V(IV) (Zhang B. et al., 2015).

### Uranium

Leachate in uranium processing areas have low but stable uranium concentrations and can contaminate water resources, groundwater and sediment (Williams et al., 2010). To solve this problem in situ, metal reducing bacteria are usually used together with the acetate feed (Vrionis et al., 2005). While uranium removal can be done by adsorption, biological reduction or membrane filtration, cathodic U(VI) reduction seems also to be a promising method. Williams et al. (2010) set an acetate fed-MFC system which consisted of a reference electrode in a uranium contaminated aquifer sediment and another electrode at the surface. This inexpensive and minimally invasive system demonstrated 10 mW/m^2^ power density during sulfate reduction and U(IV) removal (Williams et al., 2010). The removal pathway of U(IV), however, is still uncertain. The removal mechanism of the uranium may be explained as reductive immobilization of U(IV) by non-acetate oxidizing sulfate reducers (N'Guessan et al., 2008).

### Chloroethenes

Chlorinated aliphatic hydrocarbons (CAHs), which are widely used as solvents and degreasing agents, could become a huge risk due to their toxic and carcinogenic properties. These pollutants can be removed by some anaerobic bacteria which remove chlorines from CAHs by degrading them with the electrons obtained from an external electron donor or externally supplied voltage (Holliger and Schraa, 1994). An alternative version of this approach is to use insoluble electrodes to provide electrons to dechlorinating communities. Studies with two different communities (mixed culture of *dechlorinating bacteria* and pure culture of *Geobacter lovleyi*) showed that in a mixed culture, dechlorination of TCE was successfully achieved under acetate fed conditions (Aulenta et al., 2009). The formed dechlorination products were cis-DCE (83.9 ± 8.0%, on a molar basis), VC (3.5 ± 2.0%), as well as ethene and ethane (12.6 ± 7.0%). It has been proved that polarized carbon paper electrode can be used as the sole electron donor for the complete dechlorination of TCE with a mixed culture (Aulenta et al., 2009). Supplying external electron donors to the contamination zone may result in some unwanted processes and accumulate byproducts. In that case MFC with a solid electrode has a great advantage since bacterial oxidation happens in the anode and no external organic matter is added to the site (Aulenta et al., 2007).

### 2-chlorophenol

As reported in the case of chloroethens, using solid electrode as the sole electron donor is more advantageous than using soluble electron donors directly (Aulenta et al., 2009). Application of electrodes to support necessary electrons for pollutant reduction, could be used for bioremediation of chlorinated contaminants and metals (Strycharz et al., 2010). *Geobacter* is one of the typical species used for this purpose. Strycharz et al. (2010) reported that *Anaeromyxobacter dehalogenans* could also transfer electrons to 2-chlorophenol and finally dechlorinate it to phenol. In their study, acetate was initially applied (10 mM) as a substrate for *Anaeromyxobacter dehalogenans* while 80 μM 2-chlorophenol was used as an electron acceptor. The most rapid rates of dechlorination were 40 μM Cl/d (in 200 mL) which shows that bioremediation of contaminants with electrodes acting as an electron donor could be useful (Strycharz et al., 2010). More recently, dechlorination of 2-chlorophenol has also been examined by Akbulut et al. (2012). One hundred fifty micro molar 2-chlorophenol was removed with a crude laccase enzyme under optimum dechlorination conditions (Akbulut et al., 2012). There are several advantages to use solid electrodes as an electron donor for chloroethens reduction (Strycharz et al., 2010). Firstly, electrons can be effectively transferred to microorganisms for reducing the pollutant. Secondly, the electrode as electron donor can be easily applied to the site. Thirdly, if contaminants are reacted directly with electrode, unwanted reaction can be eliminated. Lastly, contaminant metals can be extracted from the electrode surface where they precipitated.

## Conclusions and future perspectives

This review summarizes the various cathodic electron acceptors that have been used in MFCs Some of these electron acceptors are also pollutants in aquatic systems. Therefore, a treatment process is also possible with MFC. A list of different cathodic electron acceptors used in MFC and the resulting power generation are summarized in the Table 1. Yet, the list is by no means exhaustive as newer electron acceptors may emerge accompanying the development of cathodic catalysts, electrode materials and solution chemistry. In the early applications of MFC, oxygen was commonly used as a terminal electron acceptor in the cathode chamber. However, in recent years, researchers are exploring more unconventional cathodic electron acceptors with an aim of improving MFC voltage potential on one hand and treating special wastewater or recovering valuable chemical on the other hand. The production of electricity with the reduction of specific electron acceptors in the cathode has promising potential in terms of bioenergy production as well as reducing the cost of special pollutant treatment (e.g., nitrogen species, persulfate, mercury, copper, chromium and perchlorate) Thus, contaminants that have high redox potential could be removed by reduction in the cathode compartment. MFC could be more efficient by using specific electron acceptors. Ferricyanide or hydrogen peroxide may be used for high power output or iron could be used to release of some valuable compounds such as phosphate from wastewaters.

Cathodic electron acceptors being used in MFCs have grown in diversity. The aim of alternative electron acceptors exploration shifted from initial high voltage output to both energy production and recalcitrant pollutant treatment or valuable chemical recovery.

Similar application of MFC configuration in the contaminated site remediation is to apply electrodes into the land and providing the voltage externally to transfer electrons to microorganisms as mentioned in Section Chloroethenes and 2-Chlorophenol. This application could provide the effective delivery of electrons. By this way, electrodes can also place to site according to the remediation requirements. On the other hand, reduced metals and other pollutants can be effectively removed from the site by precipitating on the electrode surface.

Electricity current is an indication of the microbial activity in MFC. Thus, biosensors can be developed on the basis of MFC to detect substances which may directly affect the microbial activity (i.e., BOD or toxic compounds). This process is related to activity in the anode compartment, and MFCs can also be developed as cathodic biosensors for monitoring specific pollutants in the cathode compartment according to varied redox potentials.

## Author contributions

DU: Substantial contributions to the conception or design of the work; or the acquisition, analysis, or interpretation of data for the work; IA: Drafting the work or revising it critically for important intellectual content; DU and YZ: Agreement to be accountable for all aspects of the work in ensuring that questions related to the accuracy or integrity of any part of the work are appropriately investigated and resolved; YZ: Critical revision; IA: Final approval of the version to be published.

### Conflict of interest statement

The authors declare that the research was conducted in the absence of any commercial or financial relationships that could be construed as a potential conflict of interest.
